# Supplementary material for: Distinct value computations support rapid sequential decisions
Source: Nat Commun. 2023 Nov 21;14:7573. doi: 10.1038/s41467-023-43250-x (PMC10663503; doi:10.1038/s41467-023-43250-x)
Supplement: Supplementary file 1 — Supplementary Information [file 41467_2023_43250_MOESM1_ESM.pdf]

Supplementary Information

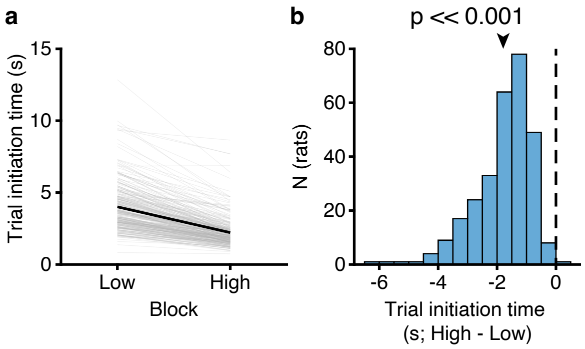

Supplementary Fig. 1: **Trial initiation time in units of seconds. a.** Trial initiation time by block ($N = 291$). Data are replotted from Fig. 1h but in units of seconds. **b.** Trial initiation time difference (high - low) across all rats. (two-tailed Wilcoxon signed-rank test, $p = 1.82 \times 10^{-49}$, $N = 291$).

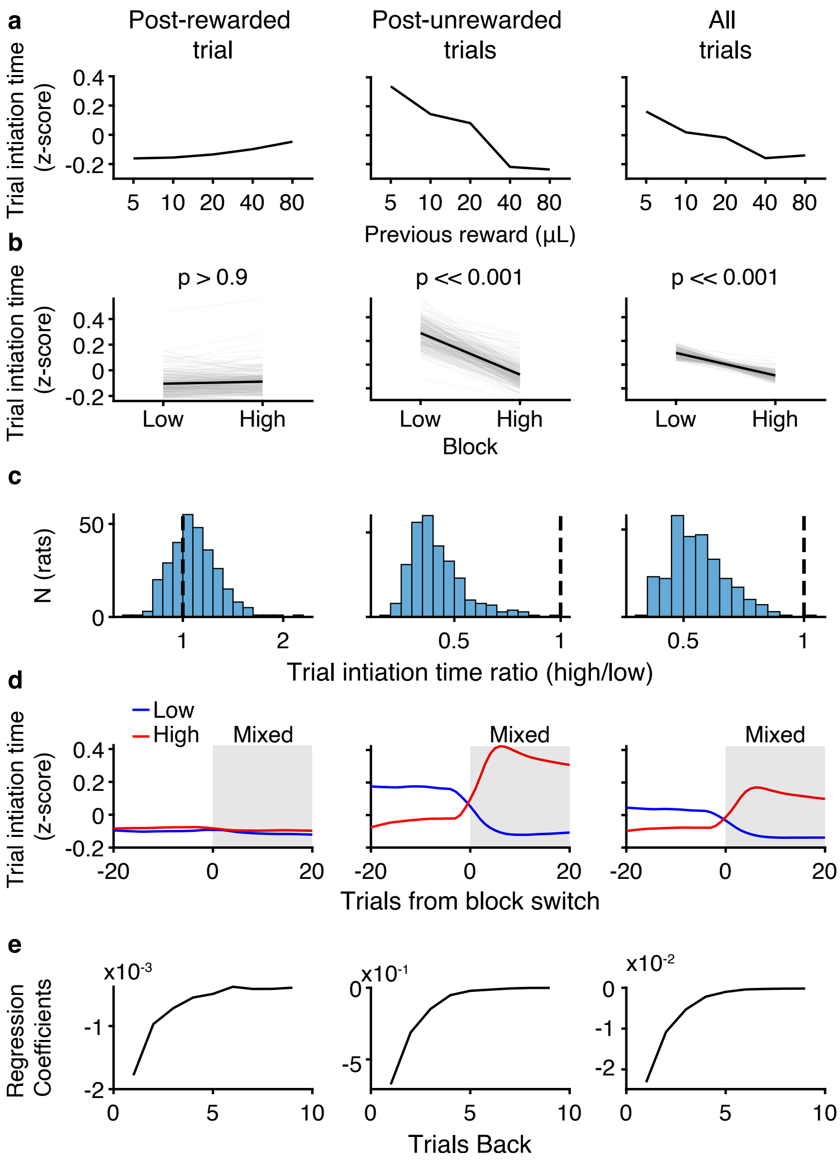

Supplementary Fig. 2: **Trial initiation times depend on previous trial outcome.** **a.** Trial initiation time by previous reward in mixed blocks for (left) post-rewarded trials, (center) post-unrewarded trials, and (right) all trial ($N = 291$). **b.** Trial initiation time averaged over block (two-tailed Wilcoxon Signed-rank test, Post-rewarded $p = 1$, Post-unrewarded $p = 9.07 \times 10^{-50}$, Post-rewarded $p = 9.17 \times 10^{-50}$, $N = 291$). **c.** Trial initiation time ratio (mean trial initiation time in high blocks/low blocks, $N = 291$). **d.** Mean change trial initiation times from low or high blocks to mixed blocks, $N = 291$. **e.** Previous trial regression coefficients in mixed blocks, $N = 291$. All error bars are mean ± S.E.M.

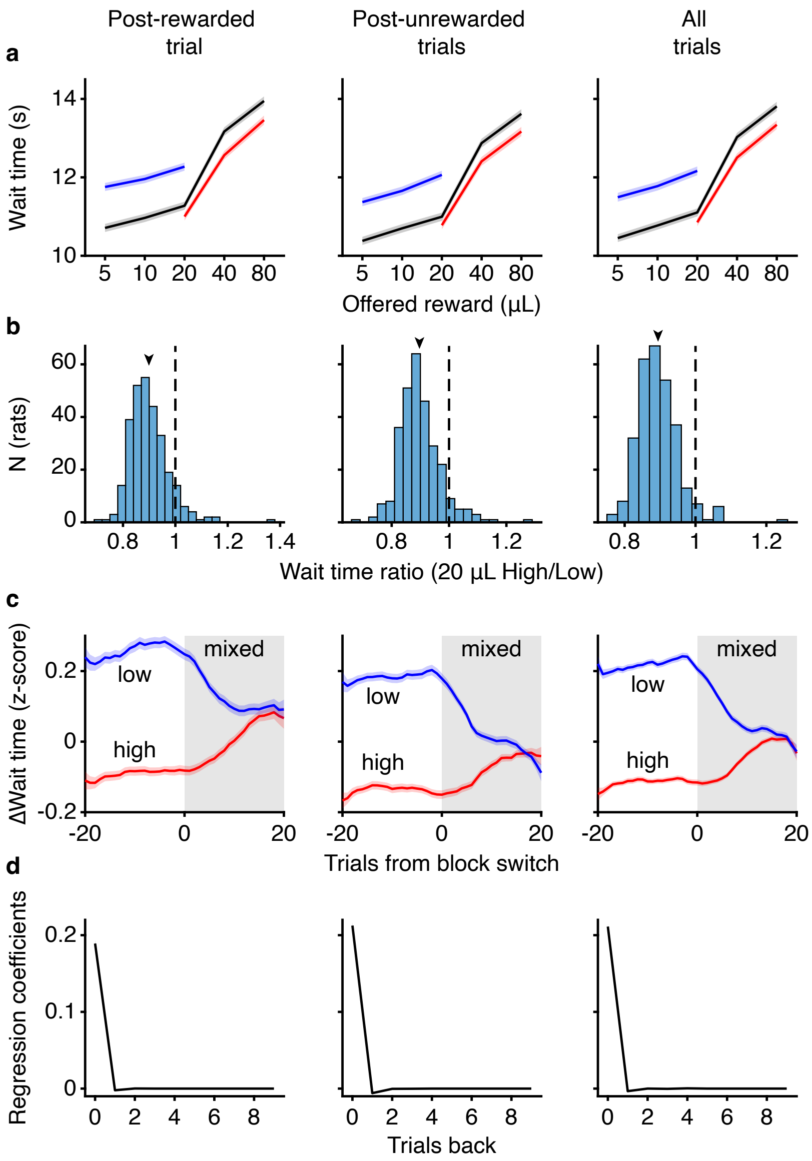

Supplementary Fig. 3: **Wait times are not affected by previous trial outcome. a.** Average wait time by volume for each block conditioned on whether the previous trial was (left) rewarded or (center) unrewarded, and (right) all trials ($N = 291$). **b.** Wait time ratios (wait time for 20 μL High/Low) across rats ($N = 291$). **c.** Wait time dynamics transitioning from low (blue) or high (red) blocks into mixed blocks ($N = 291$). **d.** Previous trial regression coefficients in mixed blocks, $N = 291$. All error bars are mean ± S.E.M.

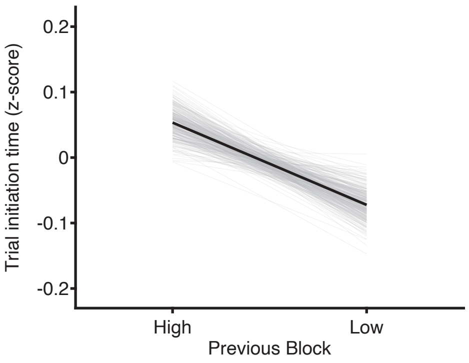

Supplementary Fig. 4: **Average trial initiation time in mixed blocks conditioned on the previous block** ($N = 291$).

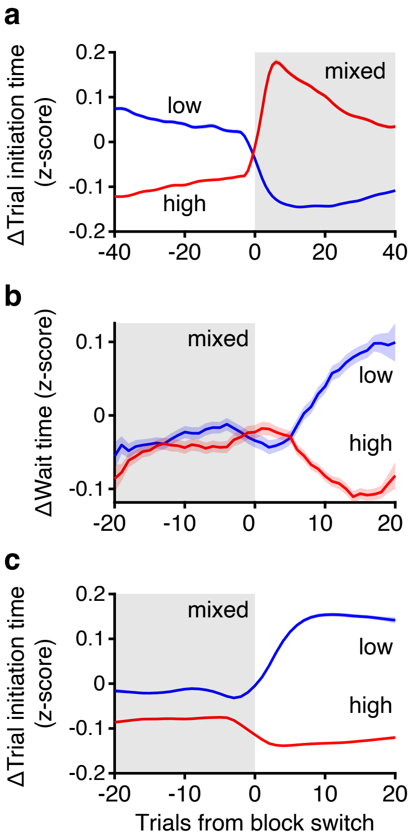

Supplementary Fig. 5: **Dynamics of wait times (top) and trial initiation times (bottom) at transitions from mixed to high (red) or low (blue) blocks. a.** Data are replotted from Fig. 2b, but with expanded x-axis limits. Trial initiation times still maintain contrast effects 40 trials into mixed blocks. **b.** Wait time transitions from mixed to high (red) and low (blue) blocks. **c.** Trial initiation time transitions from mixed to high (red) and low (blue). Block labels refer to the block at trial 0 after the mixed block. Colors are flipped relative to Fig. 2b because a current low block (blue here) is always preceded by a high block (red in Fig. 2b). $N = 291$. All error bars are mean ± S.E.M.

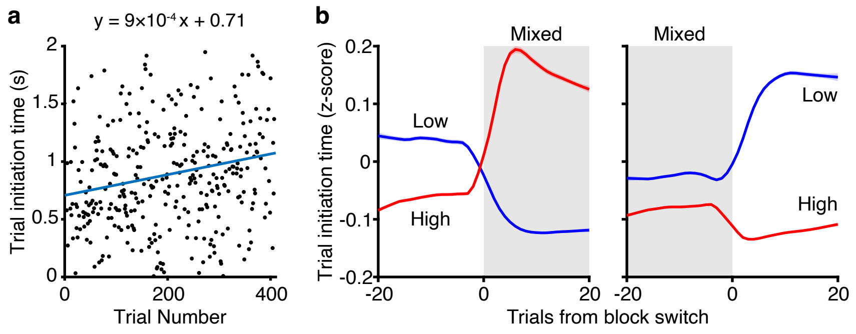

Supplementary Fig. 6: **Satiety effects for trial initiation time are modest and do not qualitatively affect results a.** Trial initiation time for an example session as a function of trial number. Line is least-squares regression. **b.** Trial initiation times block transition plots without detrending. Results are qualitatively similar to Fig. 2. $N = 291$. All error bars are mean ± S.E.M.

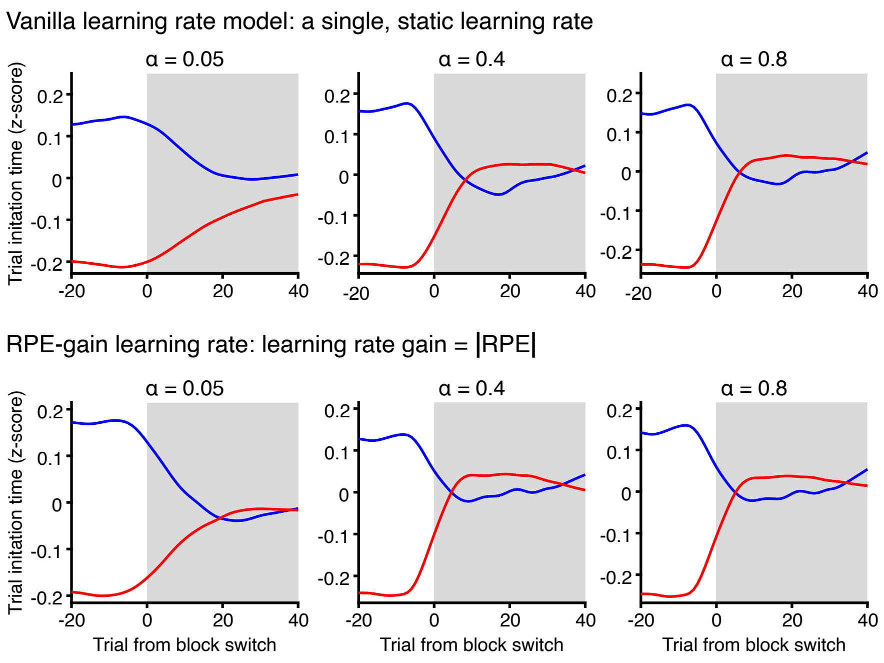

Supplementary Fig. 7: **Alternative retrospective models fail to capture both fast and slow trial initiation time dynamics at block transitions.** Trial initiation time model transitions from low (blue) or high (red) blocks to mixed blocks. Top: A vanilla learning rate model with a single, static learning rate. Bottom: a dynamic learning rate model where learning rate gain is equal to the unsigned RPE of that trial. Data are smoothed by a moving mean with window of 20 trials. $N = 50$ simulations. All error bars are mean ± S.E.M.

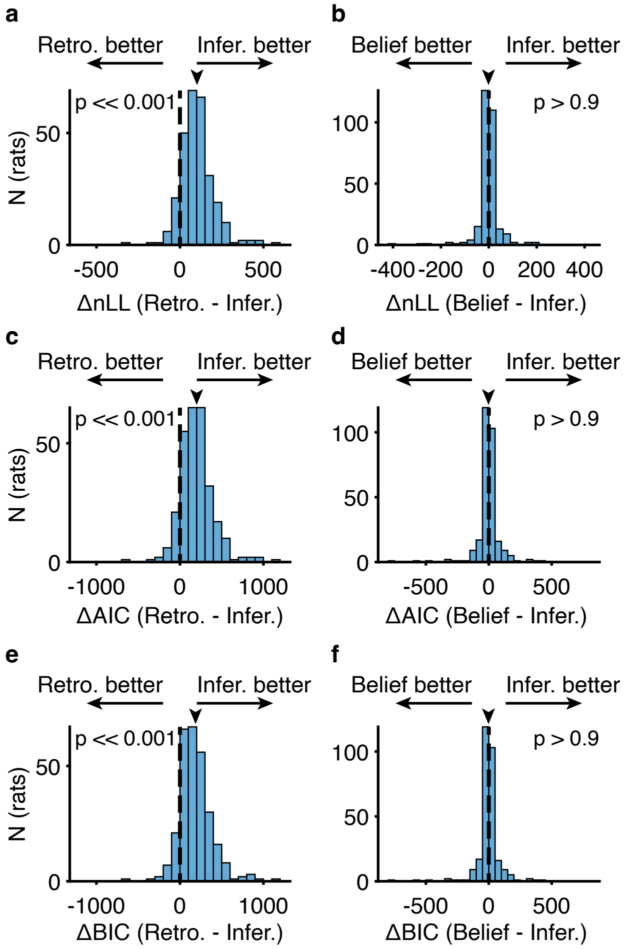

Supplementary Fig. 8: **Model comparison for wait times favors inferential over retrospective model, but does not distinguish between inferential and belief state models. a-b.** Cross-validated negative log-likelihood comparing inferential model and (a., $p = 6.58 \times 10^{-40}$) retrospective or (b., $p = 1.00$) belief state model. **c-d.** Akaike information criterion (AIC) comparing inferential model and (c., $p = 1.89 \times 10^{-39}$) retrospective or (d., $p = 1.00$) belief state model. **e-f.** Bayesian information criterion (BIC) comparing inferential model and (e., $p = 1.07 \times 10^{-37}$) retrospective or (f., $p = 1.00$) belief state model. For each, two-tailed Wilcoxon signed-rank test, $N = 291$

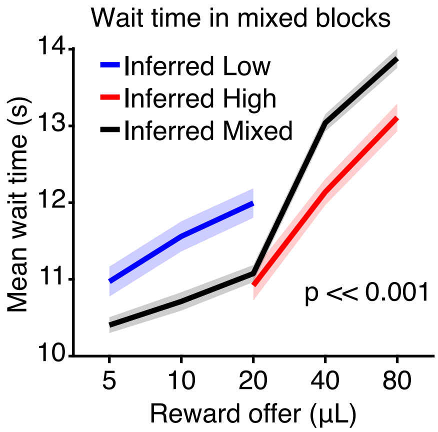

Supplementary Fig. 9: **Inferential model identifies mistaken inferences during mixed blocks across rats.** Average wait time curves conditioned by model-inferred block in mixed blocks only in held-out test set across rats.

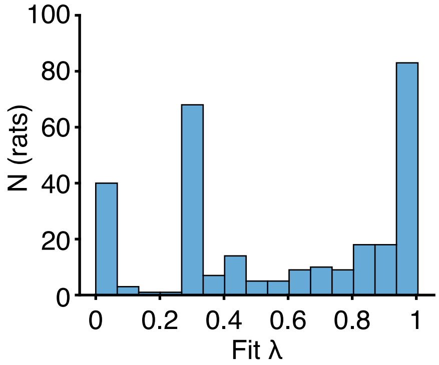

Supplementary Fig. 10: **Sub-optimal inferential model with lambda.** Distribution of λ fit over rats ($N = 291$).

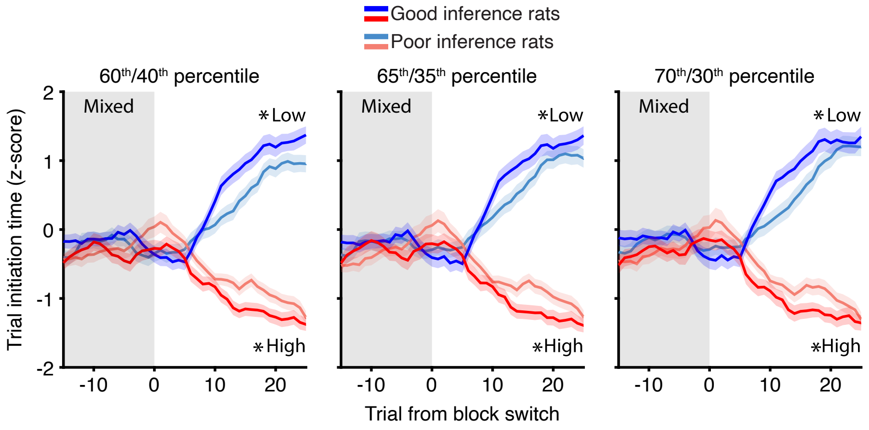

Supplementary Fig. 11: **Differential wait time dynamics based on λ from sub-optimal Bayes model are robust across a range of percentiles.** All error bars are mean ± S.E.M.

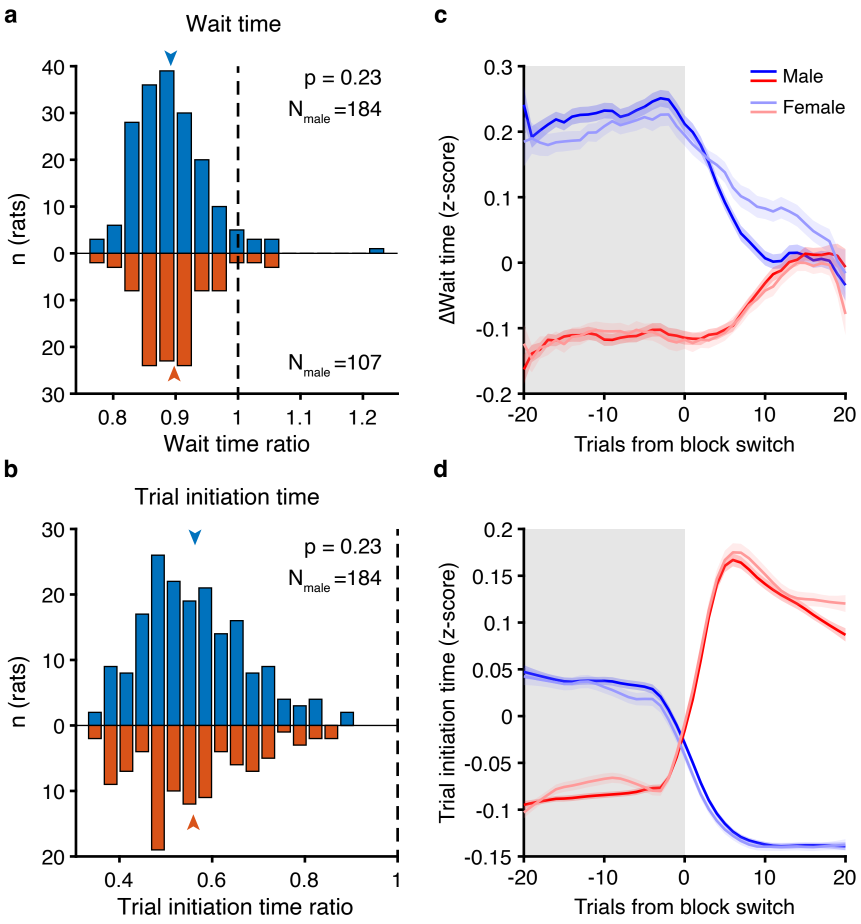

Supplementary Fig. 12: **Males and females have comparable behavior**. **a-b.** wait time ratios (a.) and trial initiation time ratios (b). Wait time $p = 0.23$, Wilcoxon Rank-sum test, $N = 184$ males, 107 females. Trial initiation time $p = 0.59$, Wilcoxon Rank-sum test, $N = 184$ males, 107 females. **c-d.** wait time (a.) and trial initation time (b.) dynamics at block transitions $N = 184$ males, 107 females

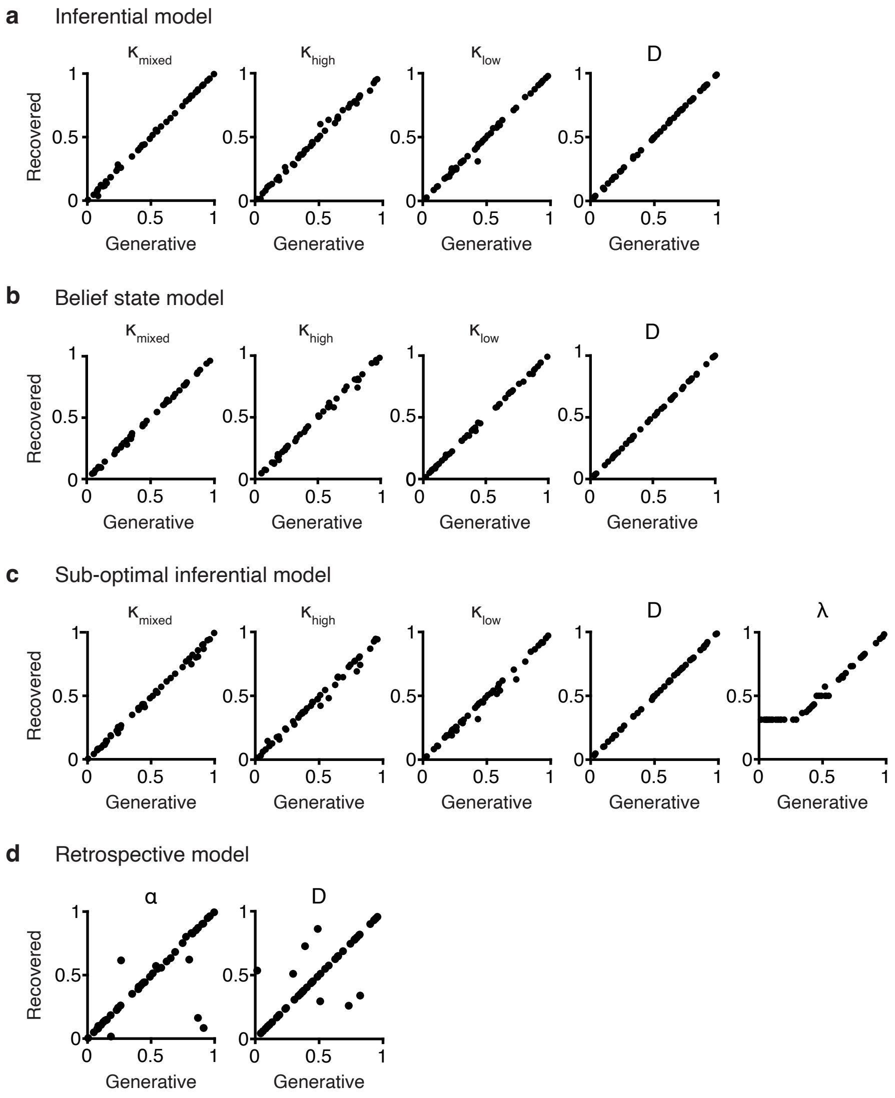

Supplementary Fig. 13: **Models are able to recover generative parameters.** $N = 48$ randomly generated parameter sets for **a.** inferential model, **b.** belief state model, **c.** sub-optimal inferential model, **d.** retrospective model

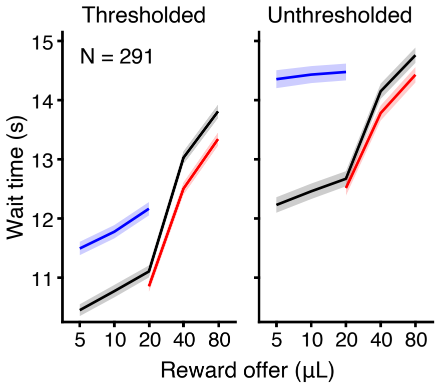

Supplementary Fig. 14: **Wait time curves without threshold (right) have qualitatively similar context effects, but longer average wait times.** Wait times one standard deviation above the pooled session mean were excluded for most analyses in this study (left). Including all wait times preserved the contextual effects, but resulted in longer average wait times, as the mean is particularly sensitive to outliers. Outlier wait times tended to occur in low blocks, likely due to attentional or motivational lapses. Therefore, the main difference between the thresholded and unthresholded data is that the wait time curves in low blocks are both flatter and longer in the unthresholded data. All data are mean ± S.E.M.
