# Supplementary material for: Distinct value computations support rapid sequential decisions
Source: Nat Commun. 2023 Nov 21;14:7573. doi: 10.1038/s41467-023-43250-x (PMC10663503; doi:10.1038/s41467-023-43250-x)
Supplement: Supplementary file 3 — Reporting Summary [file 41467_2023_43250_MOESM3_ESM.pdf]

nature portfolio

Corresponding author(s): Christine Constantinople

Last updated by author(s): Oct 22, 2023

Reporting Summary

Nature Portfolio wishes to improve the reproducibility of the work that we publish. This form provides structure for consistency and transparency in reporting. For further information on Nature Portfolio policies, see our Editorial Policies and the Editorial Policy Checklist.

Statistics

For all statistical analyses, confirm that the following items are present in the figure legend, table legend, main text, or Methods section.

n/a	Confirmed	
☐	☒	The exact sample size (n) for each experimental group/condition, given as a discrete number and unit of measurement
☒	☐	A statement on whether measurements were taken from distinct samples or whether the same sample was measured repeatedly
☐	☒	The statistical test(s) used AND whether they are one- or two-sided<br>*Only common tests should be described solely by name; describe more complex techniques in the Methods section.*
☐	☒	A description of all covariates tested
☒	☐	A description of any assumptions or corrections, such as tests of normality and adjustment for multiple comparisons
☐	☒	A full description of the statistical parameters including central tendency (e.g. means) or other basic estimates (e.g. regression coefficient) AND variation (e.g. standard deviation) or associated estimates of uncertainty (e.g. confidence intervals)
☐	☒	For null hypothesis testing, the test statistic (e.g. F, t, r) with confidence intervals, effect sizes, degrees of freedom and P value noted<br>*Give P values as exact values whenever suitable.*
☒	☐	For Bayesian analysis, information on the choice of priors and Markov chain Monte Carlo settings
☒	☐	For hierarchical and complex designs, identification of the appropriate level for tests and full reporting of outcomes
☐	☒	Estimates of effect sizes (e.g. Cohen's d, Pearson's r), indicating how they were calculated

Our web collection on statistics for biologists contains articles on many of the points above.

Software and code

Policy information about availability of computer code

Data collection	Data was collected using custom Matlab code (v2023a) running on a BPod State Machine (r2, Sanworks).
Data analysis	Data was analyzed with code written in Matlab (v2023a) that is publicly available at https://github.com/constantinoplelab/published/tree/main/rat_behavior. This information has been included in the Data and Code Availability section of the paper.

For manuscripts utilizing custom algorithms or software that are central to the research but not yet described in published literature, software must be made available to editors and reviewers. We strongly encourage code deposition in a community repository (e.g. GitHub). See the Nature Portfolio guidelines for submitting code & software for further information.

Data

Policy information about availability of data

All manuscripts must include a data availability statement. This statement should provide the following information, where applicable:

- Accession codes, unique identifiers, or web links for publicly available datasets
- A description of any restrictions on data availability
- For clinical datasets or third party data, please ensure that the statement adheres to our policy

Data is publicly available on Zenodo at https://zenodo.org/records/10031483. This information has been included in the Data and Code Availability section of the paper.

Research involving human participants, their data, or biological material

Policy information about studies with human participants or human data. See also policy information about sex, gender (identity/presentation), and sexual orientation and race, ethnicity and racism.

Reporting on sex and gender	Data was collected for both male and female rats. We found no differences in their behavior (Supplemental Figure 12), so all figures include data from both male and female rats.
Reporting on race, ethnicity, or other socially relevant groupings	N/A
Population characteristics	N/A
Recruitment	N/A
Ethics oversight	New York University Animal Welfare Committee (UAWC #2021-1120)

Note that full information on the approval of the study protocol must also be provided in the manuscript.

Field-specific reporting

Please select the one below that is the best fit for your research. If you are not sure, read the appropriate sections before making your selection.

☒ Life sciences ☐ Behavioural & social sciences ☐ Ecological, evolutionary & environmental sciences

For a reference copy of the document with all sections, see nature.com/documents/nr-reporting-summary-flat.pdf

Life sciences study design

All studies must disclose on these points even when the disclosure is negative.

Sample size	We collected a large sample size (N = 291) in order to capture as much behavioral variability as possible across a large population of rats.
Data exclusions	We did not include behavioral sessions where wait time and log(reward) were not linearly related, for at least 2 consecutive sessions. Wait times were removed on a session-by-session basis if they were greater than 2 standard deviations above the mean. Trial initiation times were excluded if they were above the 99th percentile of cumulative trial initiation times.
Replication	All rats received the exact same training protocol. Where applicable, we plotted data from individual rats, to confirm that the results were generally reproduced across the population of rats.
Randomization	For Figures 1-4, there were no experimental manipulations so randomization was not necessary. For Figure 5, rats were randomly selected by drawing a small subset from the currently running rats. Their data was similar to the population data collected thus far.
Blinding	For Figures 1-4, there were no experimental manipulations so blinding was not necessary. For Figure 5, experimenters were not blinded, but all data was processed using a common pipeline developed for Figures 1-4 before the pre-initiation cue experiments were performed.

Reporting for specific materials, systems and methods

We require information from authors about some types of materials, experimental systems and methods used in many studies. Here, indicate whether each material, system or method listed is relevant to your study. If you are not sure if a list item applies to your research, read the appropriate section before selecting a response.

Materials & experimental systems

n/a	Involved in the study	
☒	☐	Antibodies
☒	☐	Eukaryotic cell lines
☒	☐	Palaeontology and archaeology
☐	☒	Animals and other organisms
☒	☐	Clinical data
☒	☐	Dual use research of concern
☒	☐	Plants

Methods

n/a	Involved in the study	
☒	☐	ChIP-seq
☒	☐	Flow cytometry
☒	☐	MRI-based neuroimaging

Animals and other research organisms

Policy information about studies involving animals; ARRIVE guidelines recommended for reporting animal research, and Sex and Gender in Research

Laboratory animals	Long-evans rats (184 male, 107 female) between ages of 6 and 24 months were used for this study (Rattus norvegicus)
Wild animals	This study did not involve wild animals.
Reporting on sex	Data was collected for both male and female rats. We found no differences in their behavior (Supplemental Figure 12), so all figures include data from both male and female rats.
Field-collected samples	This study did not contain samples collected from the field.
Ethics oversight	Animal use procedures were approved by the New York University Animal Welfare Committee (UAWC #2021-1120) and carried out in accordance with National Institutes of Health standards.

Note that full information on the approval of the study protocol must also be provided in the manuscript.

Plants

Seed stocks	*Report on the source of all seed stocks or other plant material used. If applicable, state the seed stock centre and catalogue number. If plant specimens were collected from the field, describe the collection location, date and sampling procedures.*
Novel plant genotypes	*Describe the methods by which all novel plant genotypes were produced. This includes those generated by transgenic approaches, gene editing, chemical/radiation-based mutagenesis and hybridization. For transgenic lines, describe the transformation method, the number of independent lines analyzed and the generation upon which experiments were performed. For gene-edited lines, describe the editor used, the endogenous sequence targeted for editing, the targeting guide RNA sequence (if applicable) and how the editor was applied.*
Authentication	*Describe any authentication procedures for each seed stock used or novel genotype generated. Describe any experiments used to assess the effect of a mutation and, where applicable, how potential secondary effects (e.g. second site T-DNA insertions, mosiacism, off-target gene editing) were examined.*
